# Comparative Study on Morphology, Flavor, and Taste Profile, and Descriptive Sensory Analysis of Conventional Cream Cheese and Non‐Dairy Cream Cheese

**DOI:** 10.1002/fsn3.72301

**Published:** 2026-09-02

**Authors:** Hiran Janpeng, Natthanicha Banpacha, Gerry Renaldi, Rajnibhas Sukeaw Samakradhamrongthai

**Affiliations:** ^1^ Division of Product Development Technology, Faculty of Agro‐Industry Chiang Mai University Chiang Mai Thailand; ^2^ Division of Food Science and Technology, Faculty of Agro‐Industry Chiang Mai University Chiang Mai Thailand; ^3^ INterdisciplinary and Food Product Development for Wellness Research Unit (INFRU), Multidisciplinary Research Institute (MDRI) Chiang Mai University Chiang Mai Thailand; ^4^ Sensory Evaluation and Consumer Testing Unit, Faculty of Agro‐Industry Chiang Mai University Chiang Mai Thailand

**Keywords:** cream cheese, microstructure, sensory evaluation, volatile compound, watermelon seed

## Abstract

This study developed and optimized watermelon seed–based cream cheese (WCC) as a plant‐based alternative to dairy cream cheese and compared its microstructure, volatile compounds, taste profiles, amino acid composition, and sensory characteristics with commercial vegan cream cheese (VC) and dairy cream cheese (DC). Microstructural analysis by scanning electron microscopy showed progressive network development from the prototype (P1) to the optimized formulation (P3), with increased aggregation and porosity associated with the incorporation of hydrocolloids, salt, and maltodextrin. GC–E‐Nose analysis identified 30 volatile compounds across seven chemical groups and revealed significant differences among samples. P1 showed high levels of ethanol and aldehydes related to natural coagulation and lipid oxidation, whereas P2 and P3 showed reduced volatile intensities due to matrix stabilization and acidification. Dairy cream cheese exhibited characteristic fermentation‐related volatiles, while VC showed the lowest volatile complexity. Electronic tongue analysis demonstrated significant differences in basic taste attributes among samples, with P3 showing higher saltiness and moderate sourness, shifting its taste profile toward VC. Amino acid analysis showed the highest contents in DC, whereas WCC samples were characterized by arginine‐rich profiles and lower essential amino acid levels. Sensory descriptive analysis and PCA indicated that formulation optimization reduced seed‐derived nutty and grain notes and increased milk‐like attributes in P3. These findings demonstrate that formulation optimization improves the structural integrity and sensory similarity of WCC to commercial cream cheese.

## Graphical abstract

Systematic comparison of dairy cream cheese, commercial non‐dairy cheese, and plant‐based prototypes (P1–P3). The optimized formulation (P3) exhibited a compact microstructure (SEM), reduced beany off‐flavors (PCA), balanced taste attributes, and improved overall acceptability approaching commercial dairy standards.

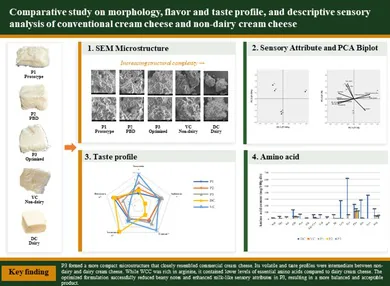

## 1 Introduction

Cream cheese is a soft, unripened acid‐coagulated dairy product characterized by its smooth texture, mild acidity, and high fat content. It is widely consumed as a spread, ingredient in sauces, dips, and baked goods, making it one of the most versatile dairy products in both sweet and savory applications (Wendin 2000; Craig et al. 2022). The global cream cheese market was valued at USD 9.1 billion in 2025 and is projected to reach USD 11.3 billion by 2035 (Future Market Insights 2025). Despite its broad appeal, dairy‐based cream cheese cannot be consumed by a significant portion of the global population. Lactose intolerance affects more than 65% of adults worldwide and reaches up to 90% in East Asian populations (Anguita‐Ruiz et al. 2020). Milk protein allergy, though less prevalent, also presents clinical significance in sensitive individuals (Høst 1994). With cream cheese containing 6%–8% lactose, even modest consumption can trigger adverse gastrointestinal symptoms in intolerant consumers (Sainani et al. 2004). Additionally, increasing consumer interest in vegan and plant‐based diets, driven by environmental sustainability and health concerns, has expanded demand for dairy‐free alternatives. The global vegan food market reached USD 23.31 billion in 2020 and is projected to grow to USD 61.35 billion by 2028 (Fortune Business Insights 2024). These combined factors have motivated the development of plant‐based cream cheese analogues capable of replicating the physicochemical and sensory properties of the dairy counterpart.

Plant‐based cream cheese is commonly formulated from soy protein, tree nuts, or coconut‐derived ingredients. However, soy, peanuts, and tree nuts are among the most prevalent food allergens and are associated with severe immunological responses (Cordle 2004; Midun et al. 2021). Tree nut allergies have increased globally over the past decade (Messina and Venter 2020), and cross‐contamination risks in shared manufacturing facilities further limit the accessibility of these products to allergic consumers (Taylor and Baumert 2010). Watermelon ( *Citrullus lanatus* ) seeds have been identified as a promising allergen‐free alternative protein source for plant‐based food development. Asia produces more than 80% of the world's watermelon, with China alone accounting for 67% of global output (Dube et al. 2021). Seeds constitute approximately 3%–4% of the fresh‐cut fruit by weight, generating millions of tons of agro‐waste annually (Tarazona‐Díaz et al. 2011). Watermelon seeds are nutritionally dense, containing 22%–28% protein, over 27%–44% unsaturated fatty acids (predominantly linoleic and oleic acids), and notable levels of minerals including magnesium, potassium, and phosphorus (Benmeziane and Derradji 2023). Their protein fraction is primarily composed of globulins rich in arginine and glutamic acid, which enables emulsification at the oil–water interface and the formation of stable protein networks around fat droplets (Hahn et al. 2025; El‐Adawy and Taha 2001). The high unsaturated fat content promotes smaller droplet sizes and a viscoelastic three‐dimensional network that contributes to spreadability and textural resilience (Kowalska et al. 2022; Zhang, Li, et al. 2022). The use of watermelon seeds as a cream cheese base simultaneously addresses allergen safety concerns, utilizes agricultural by‐products, and meets the nutritional requirements of plant‐based cheese formulation.

The physicochemical quality of cream cheese is evaluated through multiple complementary analytical approaches. Microstructural analysis by scanning electron microscopy (SEM) has been widely applied to characterize the protein–fat network of cream cheese. Dairy cream cheese is reported to exhibit a typical corpuscular microstructure consisting of fat globule clusters stabilized by protein aggregates at the fat‐water interface (Mehta 2018). The compactness of this network has been shown to correlate positively with product hardness and gel strength, while the addition of hydrocolloids such as gums produces distinct “honeycomb”‐like structural features associated with altered textural properties (Ong et al. 2020). The volatile compound profile of cream cheese is primarily generated through glycolysis, proteolysis, and lipolysis during fermentation and storage. These biochemical processes produce alcohols, aldehydes, ketones, esters, and organic acids, which collectively define the characteristic aroma of cream cheese (Wang et al. 2022). Diacetyl and acetoin, derived from citrate metabolism by lactic acid bacteria, are recognized as key contributors to the buttery and cream‐like aroma of dairy cream cheese (Mallia et al. 2008), while aldehydes arising from lipid oxidation are typically present at lower concentrations as they are converted to acids or alcohols during maturation (Frankel 1983). The taste profile of cream cheese is governed primarily by non‐volatile compounds, including free amino acids, organic acids, short‐chain fatty acids, and mineral salts (Chen et al. 2021). Free amino acids released through proteolysis serve as precursors to flavor‐active volatile compounds and directly contribute to umami, bitter, and kokumi taste attributes (Li‐Chan and Cheung 2010). In dairy cheese, glutamic acid, leucine, phenylalanine, lysine, and valine have been consistently reported as the predominant free amino acids, with essential amino acids accounting for approximately 40% of total amino acid content (Garbowska et al. 2020). In plant‐based cream cheese, the amino acid profile is determined by the source protein; watermelon seed protein is characterized by a high arginine content and a comparatively lower level of essential amino acids relative to dairy protein (Hahn et al. 2025). These differences in protein composition between dairy and plant‐based sources are expected to produce distinct volatile and taste profiles, necessitating a comprehensive comparative physicochemical characterization.

Therefore, the main objective of this study was to develop a plant‐based cream cheese analogue using watermelon seed ( *Citrullus lanatus* ). A Plackett–Burman design was first applied to screen seven formulation variables: watermelon seed milk, κ‐carrageenan, salt, citric acid, xanthan gum, guar gum, and maltodextrin to identify those with significant effects on physicochemical and sensory properties. The significant variables were subsequently optimized using a D‐optimal mixture design (11 runs, 3 center points) with response surface methodology (RSM), in which watermelon seed milk (74%), salt (3%), and maltodextrin (9%) were identified as the key factors with high model fit (*R* ^2^ = 0.73–0.99, *p* < 0.05). The resulting product was evaluated for its physicochemical properties, amino acid profile, and microstructure using scanning electron microscopy (SEM). Additionally, its sensory profile was characterized through descriptive analysis, electronic nose (E‐nose), and electronic tongue (E‐tongue) to compare its quality with both commercial vegan and dairy‐based cream cheeses.

## 2 Materials and Methods

### 2.1 Raw Materials and Ingredients

All raw materials were food‐grade and purchased in Thailand. Decorticated seeds of *Citrullus lanatus* and κ‐carrageenan were supplied by Chiang Mai Bakermart (Chiang Mai, Thailand). Food‐grade salt (Prung Thip brand) and citric acid (McGarrett brand) were purchased from Rimping Supermarket, Chiang Mai. Xanthan gum, guar gum, and maltodextrin (Krungthepchemi, Bangkok, Thailand) were obtained from Union Science Co. Ltd.

### 2.2 Chemicals and Reagents

Analytical‐grade chemicals were used throughout the study. A standard mixture of n‐alkanes ranging from hexene to hexadecane was purchased from Restek (Centre County, PA, USA). Hydrochloric acid (0.1 mol/L) and sodium chloride solution (0.1 mol/L) were obtained from AnalytiChem Belgium NV (Zedelgem, Belgium). Monosodium L‐glutamate monohydrate was supplied by Glentham Life Sciences (Wiltshire, United Kingdom). Purified water was prepared using a Millipore water purification system (Millipore, Bedford, MA, USA). A standard solution of amino acids mixture (Type H) containing 17 common amino acids—Asp (aspartic acid), Thr (threonine), Ser (serine), Glu (glutamic acid), Pro (proline), Gly (glycine), Ala (alanine), Cys (cysteine), Val (valine), Met (methionine), Ile (isoleucine), Leu (leucine), Tyr (tyrosine), Phe (phenylalanine), His (histidine), Lys (lysine), and Arg (arginine)—was purchased from Wako Pure Chemical Corporation, Osaka, Japan. L‐leucine (reagent grade) was purchased from Sigma‐Aldrich, Singapore. Phthaldialdehyde for fluorometric detection and N‐acetyl‐L‐cysteine for post‐column derivatization were obtained from Merck (Darmstadt, Germany). Sodium citrate salts for preparing mobile phases (pH 3.23 and pH 10.0) and sodium hydroxide solution (0.2 M) were sourced from RCI Labscan, Bangkok, Thailand. HPLC grade methanol and acetonitrile were also purchased from RCI Labscan (Bangkok, Thailand). All solutions and dilutions were prepared using deionized water generated with a Milli‐Q system (Zeneer UP 900, Seoul, Korea).

### 2.3 Preparation of WCC Product

The watermelon seed cream cheese (WCC) was prepared in three formulations: prototype (P1), Plackett–Burman (P2), and optimization (P3). For P1, the preparation followed the method of Oupathumpanont et al. (2025) and Zulkurnain et al. (2008) with slight modifications. Decorticated watermelon seeds were washed with clean water. The seeds were then heated to a temperature of 80°C for 5 min to sterilize and soften them for easier blending. After draining the water, the seeds were blended with water at a ratio of 1:5 (w/v) using a blender (EM‐ICE POWER, Sharp, Thailand). The resultant slurry was manually squeezed with a muslin cloth to obtain watermelon seed milk, while the watermelon residue was discarded. The watermelon seed milk was heated to 60°C–75°C. The formulation of WCC using various variables is shown in Table 1. In the P1 prototype, no additives were added and coagulation occurred naturally. In P2 and P3 formulations, citric acid, xanthan gum, κ‐carrageenan, guar gum, salt, and maltodextrin were added during heating according to the Plackett–Burman experimental design (P2) or the optimized levels derived from response surface methodology (P3 optimized composition: 74.0% watermelon seed milk, 3.0% salt, and 9.0% maltodextrin, with the remaining four additives maintained at their lowest levels). The experimental design of watermelon seed milk, salt, and maltodextrin based on the D‐optimal mixture design with three center points is presented in Table S1. Coagulation was carried out for 30 min while gently stirring the mixture manually at a steady, continuous pace to ensure uniform coagulation. The mixture was then allowed to rest without stirring for an additional 15 min to complete gel formation. The resultant coagulum was broken thoroughly and transferred to a mold lined with cheesecloth. The mold featured perforations on the sides and bottom. The curd was pressed to produce firm WCC and stored at 4°C for 24 h before testing. The appearance of the developed WCC formulations (P1–P3) is presented in Figure S1.

**TABLE 1:** The formulation of WCC using various variables.

Formula	Variables (%)						
	Watermelon seed milk	Salt	Maltodextrin	Citric acid	Xanthan gum	κ‐carrageenan	Guar gum
P1 (prototype)	80.0	1.0	—	5.0	—	—	—
P2 (PBD)	70.0	5.0	10.0	4.0	3.0	3.0	3.0
P3 (optimized)	74.0	3.0	9.0	4.0	3.0	3.0	3.0

### 2.4 Scanning Electron Microscopy (SEM) of WCC Product

The WCC samples were frozen at −50°C and vacuum freeze‐dried for 48 h using a Labconco FreeZone 6 Plus Freeze Dryer (Labconco Corporation, USA). The dried samples were evaluated using a scanning electron microscope (JEOL JSM‐IT200 In Touch Scope, Japan) mounted on aluminum stubs using double‐sided conductive tape and examined at 500–1000× magnification. The sample slices were coated with a gold palladium (60:40) using a sputter coater and observed under an accelerated voltage of 3 kV (Luo et al. 2025).

### 2.5 Volatile Compounds Analysis Using Gas Chromatograph–Electronic Nose (GC‐E‐Nose)

Approximately 0.3 g of sample was placed into a 10 mL headspace vial and incubated at 60°C for 10 min prior to analysis. Volatile compounds were analyzed using a Heracles II E‐Nose (Alpha M.O.S., Toulouse, France) equipped with an MXT‐5 column (280 μm × 0.5 μm). Detection was carried out using flame ionization detectors (FID). Approximately 0.3 g of each sample was incubated at 60°C for 10 min before analysis. A 4 mL portion of headspace gas was injected at a rate of 250 μL/s, and the injection temperature was set at 200°C. The trap temperature was kept at 50°C, with a split flow rate of 10 mL/min. The column oven temperature was initially set at 50°C, increased to 80°C at 1°C/s, and then raised to 250°C at 2°C/s, where it was held for 20 s. The detector temperature was maintained at 260°C.

### 2.6 Taste Profile Analysis Using Electronic Tongue (E‐Tongue)

Cream cheese samples were extracted in a 15% ethanol solution for 8 h at 4°C and then filtered through filter paper. Taste profiles were evaluated using an Astree e‐tongue system (Alpha M.O.S., Toulouse, France) equipped with a #6 sensor array. Sourness, saltiness, umami, sweetness, and bitterness were detected by the AHS, CTS, NMS, ANS, and SCS sensors, while PKS and CPS provided broader taste information. Measurements were collected over 140 s with a 1‐s acquisition interval. Signals obtained between 120 and 140 s, corresponding to the stable region, were averaged and used as the response values (Banpacha et al. 2025). The sensors were cleaned with deionized water after each run, and each sample was analyzed in triplicate.

### 2.7 Amino Acid Profile Using HPLC

The cream cheese samples were frozen at −50°C and vacuum freeze‐dried, then ground into a fine powder. A 10 g portion of the freeze‐dried powder was mixed with deionized water at a ratio of 1:10 (w/v) (100 mL) by blending with a hand blender (800 W, Philips, Thailand) for 2 min. The mixture was sonicated for 10 min at 25°C and then centrifuged at 8000 rpm for 15 min at the same temperature. The supernatant was filtrated through a Whatman No. 4 filter paper and lyophilized to obtain crude cream cheese extract. The determination of 17 amino acids was carried out based on the method of Somjai et al. (2022). Using a post‐column reaction approach. An amino acid mixture standard (Type H) containing 17 common amino acids: aspartic acid (Asp), threonine (Thr), serine (Ser), glutamic acid (Glu), proline (Pro), glycine (Gly), alanine (Ala), cysteine (Cys), valine (Val), methionine (Met), isoleucine (Ile), leucine (Leu), tyrosine (Tyr), phenylalanine (Phe), histidine (His), lysine (Lys), and arginine (Arg) was used for identification and quantification. A Shim‐pack Amino‐Na column (100 mm × 6.0 mm I.D., 5 μm; P/N: 228‐18,837‐91, Shimadzu, Japan) was employed together with a Prominence RF‐20A detector (Shimadzu, Japan). Three sodium‐based mobile phases (A, B, and C) were prepared for the analysis. Mobile phases A and B consisted of sodium citrate buffers adjusted to pH 3.23 and pH 10.0, respectively, while mobile phase C was prepared as a 0.2 M sodium hydroxide solution. The reaction reagents were prepared using phthaldialdehyde (OPA) and N‐acetylcysteine for pre‐column derivatization of amino acids. The chromatographic conditions were set at a column oven temperature of 60°C, a flow rate of 0.4 mL/min, and an injection volume of 10 μL.

### 2.8 Descriptive Sensory Analysis on Dairy and Non‐Dairy Cream Cheese

A hybrid descriptive analysis (Stone and Sidel 1993; Meilgaard et al. 2007) method was used to evaluate the WCC samples. Twenty panelists were recruited from students in the Product Development Technology Division, Faculty of Agro‐Industry, Chiang Mai University, Thailand. All panelists were between 18 and 30 years of age and included both untrained and trained individuals. Candidate panelists were screened according to ASTM (1981) guidelines. The selection procedure excluded individuals who self‐reported taste or smell impairments or any kidney or liver‐related conditions. Panelists were required to express interest in the study and be available for all test sessions. They were also required to (a) correctly identify basic taste solutions in taste recognition tests, (b) recognize or describe common odors in odor identification tasks, and (c) rank taste solutions in the correct order of intensity during the ranking test. Panelists who successfully met all criteria proceeded to the training and evaluation phases.

Twelve panelists (1 male, 11 females) attended 2 h training sessions once weekly over 8 weeks. The training protocol introduced descriptive sensory evaluation methodology and familiarized panelists with intensity rating on a 150 mm unstructured line scale using basic taste solutions. To develop a sensory vocabulary, panelists evaluated experimental watermelon seed cream cheese (WCC) samples, commercial dairy‐based cream cheese, and plant‐based cream cheese alternatives from local markets. Each panelist independently listed sensory attributes, then discussed findings as a group. This process generated twelve sensory attributes that were subsequently refined through consensus. The panel established final sensory descriptors through group agreement, defining each attribute with reference standards for selected terms. Between evaluations, panelists cleansed their palates with distilled water and unsalted crackers.

The sensory evaluation sessions were conducted in the Sensory Evaluation and Consumer Testing Unit, Department of Food Technology, Faculty of Agro‐Industry, Chiang Mai University, Thailand, where lighting, ventilation, and noise levels were controlled. The evaluation took place in individual booths within an air‐conditioned room illuminated with white light at room temperature to ensure comfort and minimize distraction. Throughout the evaluation, participants were supervised by the authors and trained research assistants to prevent communication among panelists. This study was conducted with approval from the Office of Human Research Ethics Committee, Chiang Mai University (Approval No. CMUREC 67/072).

### 2.9 Statistical Analysis

All analyses for the WCC samples were performed in triplicate. The mean and standard deviation (SD) values were calculated using SPSS Statistics 17.0 software (SPSS Inc., Chicago, IL, USA). Significant differences among samples were determined using Duncan's multiple range test at a 95% confidence level (*p* < 0.05). In addition, principal component analysis (PCA) was used to determine the relationships between the sensory attributes and the corresponding WCC samples.

## 3 Results and Discussion

### 3.1 Scanning Electron Microscopy (SEM) of WCC Product

**FIGURE 1:**
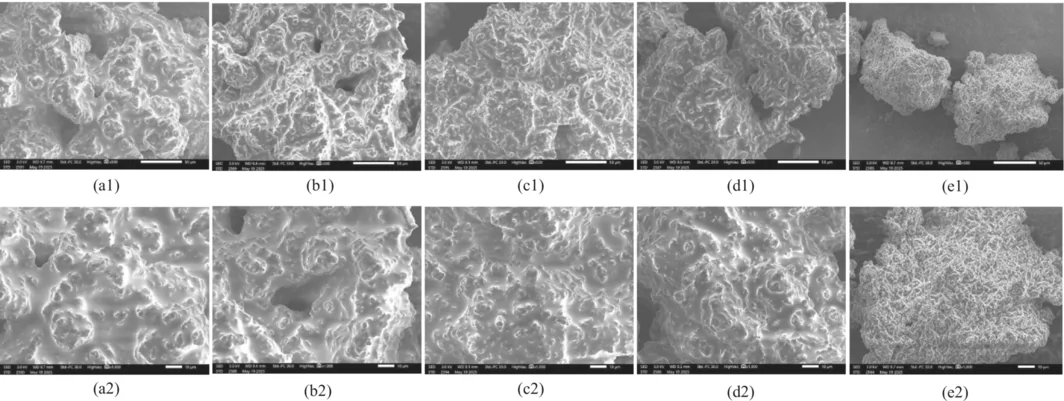
Internal structure using a scanning electron microscope (SEM) at a magnification of 500 times (a1–e1) and 1000 times (a2–e2): (a) prototype product, (b) Plackett‐burman product, (c) optimization product, (d) vegan cream cheese product from market, and (e) dairy cream cheese.

The internal microstructure of watermelon cream cheese (WCC) samples was examined using SEM at magnifications of 500× (a1–e1) and 1000× (a2–e2), as shown in Figure 1. The SEM images revealed distinct structural differences among the prototype product (P1), Plackett‐Burman product (P2), optimization product (P3), commercial vegan cream cheese (VC), and dairy cream cheese (DC). The prototype product (P1) showed a relatively smooth and homogeneous surface with minimal irregularities. This uniform morphology resulted from natural coagulation without additives, where simple protein aggregation formed a less compact network. The smooth surface may result from limited protein–protein interactions and fewer cross‐links within the protein matrix (Zhang, Wen, et al. 2022). The Plackett‐Burman formulation (P2) exhibited a more irregular and rough surface. The addition of hydrocolloids (xanthan gum, κ‐carrageenan, guar gum) and citric acid modified the protein network architecture. These characteristics may result from enhanced protein‐polysaccharide interactions that disrupted the uniform matrix observed in P1 (Turk‐Gul et al. 2023). The rougher surface suggests a more complex three‐dimensional network compared to P1. The optimization product (P3) appeared more aggregated with a rougher texture and pronounced structural features. Salt facilitated protein–protein association, while maltodextrin filled interstitial spaces, creating a denser and more compact arrangement (Ribeiro et al. 2024). The porous nature of P3 suggests improved water holding capacity, as pores function as water absorption sites. Commercial vegan cream cheese (VC) and dairy cream cheese (DC) exhibited the most coarse and rough surfaces with visible pores and intricate structural patterns. These characteristics indicate highly developed protein‐stabilizer networks typical of commercial formulations. The SEM images revealed similar microstructures between VC and DC, with noticeable pores and compact structures, suggesting that commercial vegan formulations achieve structural characteristics comparable to dairy counterparts through optimized ingredient selection. The progressive increase in surface roughness from P1 to DC correlated with network complexity and protein‐polysaccharide interactions. These results imply that appropriate concentrations of hydrocolloids, salt, and maltodextrin resulted in improved structural integrity.

### 3.2 Volatile Compounds Analysis Using Gas Chromatograph–Electronic Nose (GC‐E‐Nose)

The volatile compound profiles of watermelon cream cheese samples were analyzed using GC‐E‐Nose. A total of 30 compounds across seven chemical groups including acids, alcohols, aldehydes, ketones, furans/lactones, sulfur‐containing compounds, and terpenes were identified, as shown in Table 2. Significant differences (*p* < 0.05) in volatile composition were observed among dairy cream cheese (DC), vegan cream cheese (VC), and WCC formulations (P1, P2, and P3).

**TABLE 2:** Volatile compounds content (μg/100 g) in WCC and commercial dairy and non‐dairy cream cheese samples.

Compound (group)	DC	VC	P1	P2	P3	Sensory description
*Acids*						
Acetic acid	18.23 ± 1.47ᵃ	12.48 ± 1.21ᵇ	14.95 ± 1.34ᵇ	13.97 ± 1.11ᵇ	13.82 ± 1.18ᵇ	Sour, vinegar, pungent
Butanoic acid	42.48 ± 3.21ᵃ	17.96 ± 2.03ᵇ	28.03 ± 2.49ᶜ	24.98 ± 2.04ᶜ	22.03 ± 1.79ᵇᶜ	Rancid, cheesy, buttery
Hexanoic acid	9.51 ± 0.79ᵃ	4.02 ± 0.51ᶜ	6.52 ± 0.61ᵇ	5.81 ± 0.52ᵇ	5.03 ± 0.41ᶜ	Cheese, fatty, goat‐like
Octanoic acid	5.21 ± 0.49ᵃ	2.48 ± 0.31ᶜ	3.53 ± 0.32ᵇ	3.19 ± 0.28ᵇ	2.82 ± 0.29ᶜ	Fatty, coconut, soapy
Nonanoic acid	2.19 ± 0.21ᵃ	ND	ND	ND	ND	Fatty, waxy
*Alcohols*						
Ethanol	243.87 ± 16.12ᵇ	194.76 ± 11.97ᶜ	978.23 ± 48.12ᵃ	491.57 ± 12.93ᵇ	609.62 ± 24.91ᵇ	Alcoholic, sweet
1‐Butanol	10.68 ± 0.78ᶜ	9.08 ± 0.97ᶜ	12.23 ± 2.12ᵇ	56.79 ± 1.82ᵃ	22.49 ± 2.03ᵇ	Fruity, fermented
1‐Hexanol	1.21 ± 0.11ᶜ	0.91 ± 0.11ᶜ	3.09 ± 0.31ᵃ	2.42 ± 0.21ᵇ	1.97 ± 0.19ᵇᶜ	Green, grassy, fruity
2,3‐Butanediol	3.48 ± 0.32ᵃ	1.02 ± 0.09ᶜ	0.59 ± 0.05ᵈ	1.18 ± 0.11ᶜ	0.91 ± 0.06ᶜ	Buttery, sweet, creamy
Benzyl alcohol	1.09 ± 0.11ᵃ	ND	0.71 ± 0.06ᵇ	0.51 ± 0.06ᵇ	0.61 ± 0.05ᵇ	Floral, sweet
*Aldehydes*						
Hexanal	7.79 ± 0.61ᵇ	0.89 ± 0.09ᵈ	8.52 ± 0.69ᵃ	3.58 ± 0.42ᶜ	2.92 ± 0.31ᶜ	Grassy, green, fatty
Nonanal	2.48 ± 0.21ᵃ	0.52 ± 0.05ᶜ	1.03 ± 0.11ᵇ	0.82 ± 0.09ᵇᶜ	0.71 ± 0.06ᶜ	Waxy, citrus, sweet
(E,E)‐2,4‐Decadienal	0.95 ± 0.08ᵇ	0.41 ± 0.05ᶜ	1.38 ± 0.13ᵃ	1.04 ± 0.09ᵇ	0.86 ± 0.07ᵇ	Fried, fatty, oily
Benzaldehyde	0.81 ± 0.09ᵃ	0.21 ± 0.05ᶜ	0.29 ± 0.05ᵇᶜ	0.41 ± 0.05ᵇ	0.31 ± 0.05ᵇᶜ	Almond, sweet
*Ketones*						
2‐Heptanone	0.49 ± 0.05ᵃᵇ	0.31 ± 0.03ᶜ	0.81 ± 0.08ᵃ	0.59 ± 0.05ᵃᵇ	0.52 ± 0.05ᵇ	Cheese, fruity, floral
2‐Nonanone	0.21 ± 0.02ᵇ	ND	0.31 ± 0.03ᵃ	0.26 ± 0.02ᵃᵇ	0.19 ± 0.02ᵇ	Soapy, fruity
Acetoin	5.52 ± 0.59ᵃ	1.83 ± 0.21ᶜ	0.92 ± 0.11ᵈ	2.59 ± 0.31ᵇ	1.78 ± 0.22ᶜ	Butter, creamy, dairy
1‐Hexen‐3‐one	1.92 ± 0.21ᵃ	0.31 ± 0.05ᶜ	0.61 ± 0.05ᵇ	ND	ND	Green, grassy, fresh
1‐Octen‐3‐one	0.47 ± 0.03ᵃᵇ	0.39 ± 0.02ᵇ	0.41 ± 0.05ᵇ	0.43 ± 0.03ᵇ	0.44 ± 0.04ᵇ	Mushroom, earthy, metallic
*Furans and Lactones*						
2‐Furfural	0.31 ± 0.03ᵇ	0.19 ± 0.02ᵇ	0.51 ± 0.05ᵃ	0.41 ± 0.04ᵃᵇ	0.36 ± 0.03ᵇ	Sweet, caramel, almond
γ‐Decalactone	0.21 ± 0.02ᵇ	0.11 ± 0.01ᶜ	0.41 ± 0.04ᵃ	0.31 ± 0.03ᵃᵇ	0.26 ± 0.02ᵇ	Peach, creamy, fruity
γ‐Nonalactone	0.61 ± 0.10ᶜ	0.51 ± 0.10ᶜ	1.32 ± 0.11ᵃ	1.11 ± 0.10ᵃᵇ	1.02 ± 0.11ᵇ	Coconut, creamy, sweet
*Sulfur‐containing compounds*						
Dimethyl disulfide	0.91 ± 0.10ᵃ	0.21 ± 0.05ᶜ	0.59 ± 0.10ᵇ	0.71 ± 0.11ᵇ	0.41 ± 0.05ᶜ	Onion, sulfurous
Methional	0.21 ± 0.02ᵇ	ND	0.29 ± 0.03ᵃ	0.26 ± 0.02ᵃᵇ	0.21 ± 0.02ᵇ	Cooked potato, savory
3‐Methyl‐2‐butene‐1‐thiol	0.66 ± 0.10ᵇ	0.72 ± 0.10ᵇ	1.22 ± 0.19ᵃ	0.90 ± 0.11ᵃᵇ	0.74 ± 0.10ᵇ	Onion, sulfurous, skunky
*Terpenes*						
Limonene	0.69 ± 0.11ᶜ	0.91 ± 0.10ᶜ	2.01 ± 0.21ᵃ	1.61 ± 0.19ᵇ	1.09 ± 0.10ᶜ	Citrus, orange
β‐Pinene	1.69 ± 0.21ᵇ	1.61 ± 0.19ᵇ	2.79 ± 0.31ᵃ	2.09 ± 0.20ᵇ	1.91 ± 0.21ᵇ	Pine, woody
α‐Terpineol	0.41 ± 0.05ᵇ	0.31 ± 0.03ᵇ	0.71 ± 0.06ᵃ	0.51 ± 0.05ᵃᵇ	0.41 ± 0.05ᵇ	Floral, lilac, citrus

*Note:* Each value represents the mean ± SD. Different superscript letters in the same column show statistically significant differences (*p* < 0.05).

Abbreviations: DC, dairy cream cheese; ND, Not detected; P1, prototype; P2, plant‐based dairy (PBD); P3, optimized watermelon seed cream cheese; VC, non‐dairy cream cheese.

Three major volatile groups were recorded: alcohols, organic acids, and aldehydes. The highest concentration of ethanol (978.23 μg/100 g) was found in P1, while the highest butanoic acid (42.48 μg/100 g) was detected in DC. Additionally, hexanal was found at its highest level (8.52 μg/100 g) in P1. The highest concentrations of acids and acetoin, which are recognized as dairy fermentation markers, were shown by DC. Cheesy and fatty notes in DC were attributed to butanoic and hexanoic acids. Furthermore, buttery and creamy aromas were produced by acetoin (5.52 μg/100 g) and 2,3‐butanediol (3.48 μg/100 g) through citrate metabolism by lactic acid bacteria. Pyruvate was formed from citrate and subsequently decarboxylated to acetoin, which was then reduced to 2,3‐butanediol. Butanoic and hexanoic acids were primarily produced via microbial lipolysis through the hydrolysis of fats by native lipases to release short‐ and medium‐chain fatty acids (Luo et al. 2023). Nonanoic acid was detected only in DC, suggesting that specific dairy pathways are absent in plant‐based systems.

In sample P1, ethanol, hexanal, γ‐nonalactone, and limonene were identified as the major compounds. These patterns were found to be comparable to those reported in naturally fermented plant‐based products (Hidalgo‐Fuentes et al. 2024). The high ethanol concentration in P1 was linked to natural coagulation without the use of preservatives or pH control. The presence of aldehydes, particularly hexanal, was related to the lipid oxidation of polyunsaturated fatty acids in watermelon seeds (Zamuz et al. 2021). The reaction followed the autoxidation mechanism, where linoleic acid was oxidized to form hydroperoxides, which were cleaved via homolytic decomposition to generate hexanal as the major secondary product. Without a protective matrix or antioxidant barrier in P1 (Yang et al. 2024). Moreover, the plant origin of the seeds was reflected by the presence of terpenes (Huang and Osbourn 2019). Terpene compounds were identified as residual secondary metabolites synthesized via the mevalonate and methylerythritol phosphate pathways in watermelon seed tissue, which were carried over into the final product during processing (Xavier et al. 2023).

Lower volatile levels were observed in P2 and P3 compared to P1. A decrease in ethanol to 491.57 and 609.62 μg/100 g was recorded. A decrease in aldehydes was also seen, which was in agreement with studies by Bulat and Topcu (2020) regarding stabilized systems. It was suggested that protection against lipid oxidation was provided by hydrocolloids and maltodextrin, or that volatiles were trapped within the gel matrix (Gomes and Kurozawa 2021). The reduction was explained by two complementary mechanisms. First, a three‐dimensional gel network was formed by hydrocolloids, such as gums and maltodextrin, through hydrogen bonding and chain entanglement (Li et al. 2025). The resulting network physically encapsulated volatile molecules and reduced their diffusion toward the headspace, leading to fewer compounds being released and detected. Second, the hydrocolloid network functioned as an oxygen barrier (Gao et al. 2024), which limited oxygen diffusion toward unsaturated lipid sites and slowed hydroperoxide formation. Hexanal was generated from the oxidative cleavage of hydroperoxides (Vanoye and Favre‐Réguillon 2022), restricted oxygen access was directly linked to the lower aldehyde levels recorded in P2 and P3. Additionally, volatile compounds were entrapped by maltodextrin through hydrophobic inclusion within its glucose‐chain structure, further reducing volatile release without necessarily lowering the total compound amount in the product (Lukova et al. 2023). Such a distinction was important, as a lower detected concentration did not always indicate reduced volatile formation, but rather reflected stronger retention within the matrix.

In P2, a high level of 1‐butanol (56.79 μg/100 g) was found, possibly due to ingredient interactions. 1‐Butanol accumulation was tentatively linked to two plausible routes in a stabilized, less oxidative system: the microbial reduction of butanal and the esterase‐driven hydrolysis of formulation‐derived butanoate esters (Hussain et al. 2022). In P3, acid levels were found to be lower than DC but higher than VC, while ethanol (609.62 μg/100 g) was recorded at a higher level than both DC and VC. An acid profile was produced through a balance between limited microbial fermentation in P3 and partial volatile retention by the added hydrocolloid system.

The lowest volatile complexity was shown by VC, with low levels of acids, aldehydes, and ketones. This reduced profile was considered typical for commercial plant‐based products designed for a longer shelf life (Cirne et al. 2019). Such differences resulted from a combination of processing and formulation factors. High‐temperature processing and homogenization were typically applied to commercial plant‐based products, inactivating endogenous lipoxygenase and oxidative enzymes before significant lipid oxidation could occur, thus limiting aldehyde formation (Soendjaja and Girard 2024). Furthermore, because no active fermentation step occurred in VC, microbial pathways for short‐chain acid and ketone generation were never initiated, unlike in DC or naturally fermented P1. The absence of several compounds in VC, such as nonanoic acid and benzyl alcohol, was found to be consistent with findings by Meng et al. (2025), potentially due to processing methods that suppress volatile formation. Volatile reduction was further attributed to refining and deodorization steps commonly applied to plant‐based fat and protein ingredients. Such processes were specifically designed to strip volatile compounds from raw materials prior to formulation, thereby limiting the availability of precursor materials for subsequent volatile development in the finished product.

### 3.3 Taste Profile Analysis Using Electronic Tongue (E‐Tongue)

**FIGURE 2:**
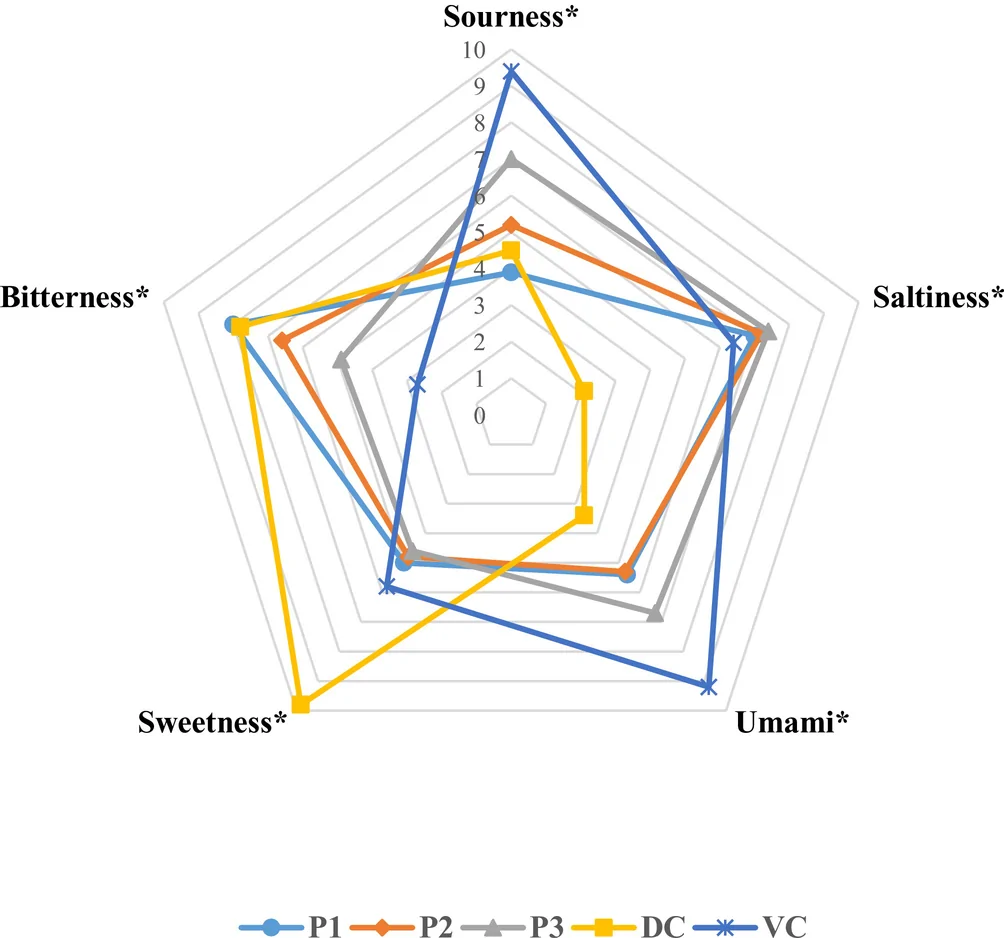
Taste profiles of WCC and commercial cream cheese samples were measured using E‐tongue analysis. DC, dairy cream cheese; P1, prototype; P2, plant‐based dairy (PBD); P3, optimized watermelon seed cream cheese; VC, non‐dairy cream cheese.

The taste profiles of WCC and commercial cream cheese samples were measured using E‐tongue analysis, as shown in Figure 2. Significant differences (*p* < 0.05) in saltiness, sourness, sweetness, umami, and bitterness were observed among samples P1, P2, P3, VC, and DC. These variations were found to be consistent with previous reports by Silva et al. (2024), where wide differences in basic taste attributes were often seen in plant‐based cream cheese formulations.

The observed taste differences may also have been influenced by protein–ion interactions in the cream cheese matrix. Since E‐tongue sensors detect taste through ionic responses, the mobility and availability of dissolved ions could have been affected by protein structure and formulation composition. Similar effects were reported by Dai et al. (2026), where plant proteins were found to influence ionic taste perception through electrostatic interactions. The mechanism mirrored the ionic basis of biological taste transduction. In human taste perception, saltiness and sourness are triggered by ${\mathrm{Na}}^{+}$ and $H^{+}$ ions interacting with specific channels (Hu et al. 2024); E‐tongue potentiometric membranes were designed to measure these same ions based on voltage signals. In protein‐containing matrices, free ${\mathrm{Na}}^{+}$ and $H^{+}$ ions were sequestered by charged side chains through electrostatic attraction, reducing the mobile ions available for sensor interaction. Consequently, variations in saltiness and sourness signals were driven by ion‐binding dynamics rather than total added salt or acid formulation (Yu et al. 2025).

The highest levels of sweetness and bitterness, along with the lowest saltiness, were recorded in DC. This pattern was comparable to observations by Sharma et al. (2025) in seed‐based dairy analogues, where sweetness and bitterness were found to increase due to carbohydrate ingredients and natural phenolic residues. Sweetness detection was governed by hydrogen bonding between dissolved carbohydrates and receptor or membrane surfaces (Sun et al. 2025), where higher residual sugar levels in DC generated stronger membrane responses. Conversely, bitterness was driven by hydrophobic and electrostatic interactions between phenolic compounds and bitterness‐sensitive membranes (Li et al. 2023), mirroring human T2R receptor activation and correlating elevated phenolic residues with higher bitterness readings. Meanwhile, lower saltiness in DC was attributed to reduced free ${\mathrm{Na}}^{+}$ availability, resulting from either lower initial salt addition or stronger ion retention within the protein matrix (Nurmilah et al. 2022).

In contrast, high sourness and umami but low bitterness were shown by VC. These results were in agreement with studies by Bulut et al. (2022), which indicated that sourness and umami may be increased by higher acidity and protein‐related components in acidified dairy alternatives. Sourness in VC was attributed to increased free $H^{+}$ availability from plant‐based acidification, directly enhancing sourness sensor response. Similarly, higher umami readings were driven by free glutamate and glutamate‐like amino acids from protein hydrolysis, which bound the sensor membrane analogously to T1R1/T1R3 receptor activation. Conversely, low bitterness was indicated by reduced hydrophobic phenolic or peptide concentrations, or by taste masking from dominant acidic and umami signals (Yang et al. 2025).

Among the samples, the highest saltiness was shown by P3, while its sourness was ranked second after VC. Lower sweetness was observed in P3 compared to VC and DC, and its umami level was also found to be lower than VC. These patterns were consistent with the effects of salt levels and formulation differences, which were reported by Shi et al. (2022) to influence the perception of basic tastes in non‐dairy. The higher saltiness detected in P3 may have been associated with greater sodium ion availability in the optimized matrix. In addition, the lower sweetness and umami intensities may have resulted from reduced release of sweet compounds and free amino acids from the protein network, as described by Diepeveen et al. (2022).

Since P3 was developed by adjusting P1 and P2, a shift toward the taste characteristics of VC was observed. Higher saltiness and moderately higher sourness were seen in P3 compared to P1 and P2, while its sweetness and bitterness were found to be at intermediate levels between DC and VC. These shifts suggested that the taste pattern of P3 was moved closer to VC through formulation adjustments, particularly regarding sourness and overall balance. Although a lower umami intensity was still seen in P3 compared to VC, the sourness and saltiness patterns were found to be more comparable to VC than those of P1 and P2.

### 3.4 Amino Acid Profile Using HPLC

**FIGURE 3:**
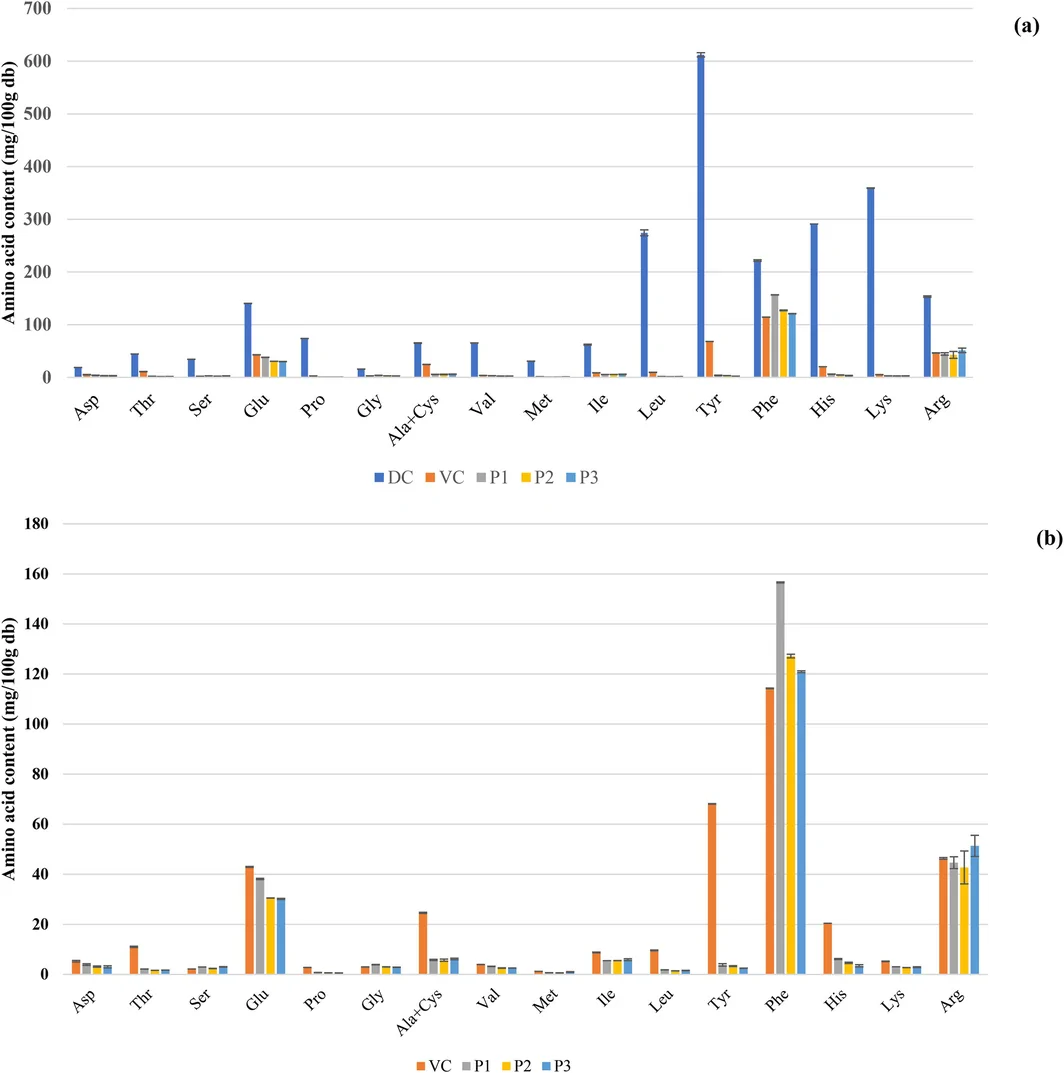
Amino acid content on different categories of cream cheese products: Aspartic acid (Asp), Threonine (Thr), Serine (Ser), Glutamic acid (Glu), Proline (Pro), Glycine (Gly), Alanine + Cystine (Ala + Cys), Valine (Val), Methionine (Met), Isoleucine (Ile), Leucine (Leu), Tyrosine (Tyr), Phenylalanine (Phe), Histidine (His), Lysine (Lys), and Arginine (Arg): (a) with DC, including DC, VC, P1, P2, and P3; and (b) without DC, including VC, P1, P2, and P3. DC, dairy cream cheese; P1, prototype; P2, plant‐based dairy (PBD); P3, optimized watermelon seed cream cheese; VC, non‐dairy cream cheese.

The amino acid profiles of the five cream cheese samples are shown in Figure 3. Representative chromatograms of the amino acid standard solution, DC, VC, and P1–P3 are presented in Figures S2–S7, respectively. Significant differences (*p* < 0.05) were observed among all samples. The highest amino acid contents were found in DC, where glutamic acid (140.26 ± 0.31 mg/100 g) and tyrosine (611.96 ± 3.94 mg/100 g) were recorded as the main components. These results were consistent with earlier reports by Chartrand et al. (2017), which stated that high levels of glutamic acid and aromatic amino acids are released from dairy proteins due to the casein structure.

In contrast, lower amino acid contents were seen in VC compared to DC. Phenylalanine (114.28 ± 0.14 mg/100 g) and arginine (46.34 ± 0.34 mg/100 g) were the most common amino acids found in this sample. Phenylalanine is classified as an aromatic amino acid and may contribute to the formation of flavor precursors involved in the development of nutty and roasted aroma notes through Maillard‐related reactions (Sun et al. 2025). The presence of phenylalanine in the developed formulation may therefore be associated with the characteristic flavor profile observed in the watermelon seed cream cheese analogue. This was in agreement with previous findings that plant‐based products usually contain lower essential amino acids depending on the raw materials (Zhang et al. 2024).

For samples P1, P2, and P3, overall low amino acid contents (0.6–156.6 mg/100 g) were observed. Arginine was found to be the major amino acid in all three samples (42.73–51.35 mg/100 g). These results were supported by Zamuz et al. (2021), who reported that watermelon seed protein is a good source of arginine. Compared to DC, lower amounts of essential amino acids, such as lysine, valine, leucine, and methionine, were detected.

Furthermore, the lower glutamic acid and tyrosine contents in P1, P2, and P3 were linked to a reduction in umami and aroma precursors. This was supported by Wang et al. (2022), who noted that plant proteins generally produce fewer flavor‐related amino acids during processing. In conclusion, the amino acid profiles in this study were determined by the natural composition of the protein sources, showing clear differences between dairy, plant‐based, and watermelon‐seed cream cheese samples.

### 3.5 Descriptive Sensory Analysis on Dairy and Non‐Dairy Cream Cheese

Sensory attributes and their definitions for WCC were developed by a trained panel, as detailed in Table 3. Based on panel discussions, 12 characteristics were selected for evaluation: yellow color, homogeneity, milk aroma, watermelon seed aroma, nut aroma, grain aroma, milk flavor, watermelon seed flavor, sour flavor, sourness, saltiness, and homogeneity during consumption. These attributes were considered to reflect the intrinsic characteristics of the watermelon seed materials as well as the added ingredients used in the prototypes. As noted in earlier work on plant‐based cheese alternatives (Dobón‐Suárez et al. 2025; Ramón et al. 2022), the presence of nutty or grain‐like notes was expected due to seed‐derived volatile compounds. Additionally, milk‐related attributes were included as references to evaluate how closely the prototype formulations approached the sensory characteristics of conventional dairy cream cheese.

**TABLE 3:** Sensory descriptors, definitions, and references for the flavor evaluation of WCC.

Attribute	Definition	Reference	Intensity
*Appearance*			
Yellow color	The intensity of yellow color from light to yellow	Color card paper measure with CIE (L*, a*, b*) CIELab (177.14, −21.56, 94.48) CIELab (97.00, 1.35, 6.95) CIELab (97.14, −21.56, 94.48)	2.0 6.0 13.0
Homogeneity	Degree of to which the sample feels smooth and free of lumps/particulates as opposed to lumpy, rough, grainy, gritty, and/or sandy	Rice flour (New grade) 85% rice flour solution 55% rice flour solution 35% rice flour solution	4.0 8.0 13.0
*Aroma*			
Milk aroma	The sweet aromatics associated with vegan milk products	Oat milk (Oatly oat milk) 50% Oat milk solution 100% Oat milk	6.0 12.0
Watermelon seed aroma	The slightly sweet, musty and nutty aromatics associated with watermelon seed	Raw watermelon seed 10 g raw watermelon seed 20 g raw watermelon seed 30 g raw watermelon seed	3.5 6.0 10.0
Nut aroma	A slight musty, sweet and brown aromatic associated with nuts	Raw peanut 5 g ground raw peanut 10 g ground raw peanut 25 g ground raw peanut	2.5 5.5 8.0
Grain aroma	The slightly pungent and nutty aromatics associated with grains	Ground Job's tears 5 g ground Job's tears 10 g ground Job's tears 25 g ground Job's tears	2.5 5.0 10.0
*Flavor*			
Milk flavor	The slightly sweet and nutty aromatics associated with vegan milk products	Oat milk (Oatly oat milk) 3 Oat milk drops 5 Oat milk drops 9 Oat milk drops	2.5 5.0 7.0
Watermelon seed flavor	The slightly earthy, musty, nutty and sweet aromatics associated with watermelon seed	Watermelon seed milk 25% watermelon seed milk solution 50% watermelon seed milk solution	6.0 12.0
Sour flavor	Sour flavor such as lime, lactic acid and citric acid and slightly sweet	Lime juice flavor 10% lime juice solution 20% lime juice solution 30% lime juice solution	3.5 7.0 10.0
*Taste*			
Sour	Fundamental taste sensation of which citric acid is typical	Citric acid solution 0.05% citric acid solution 0.08% citric acid solution 0.15% citric acid solution	2.0 5.0 10.0
Salt	Fundamental taste sensation of which sodium chloride is typical	Sodium chloride solution 0.2% sodium chloride solution 0.35% sodium chloride solution 0.5% sodium chloride solution	2.5 5.0 8.5
*Mouth‐feel*			
Homogeneity (during consumption)	Degree of to which the sample feels smooth and free of lumps/particulates as opposed to lumpy, rough, grainy, gritty, and/or sandy during mastication.	All purpose Flour (Kite) 50% all purpose flour solution 30% all purpose flour solution	10.0 15.0

**FIGURE 4:**
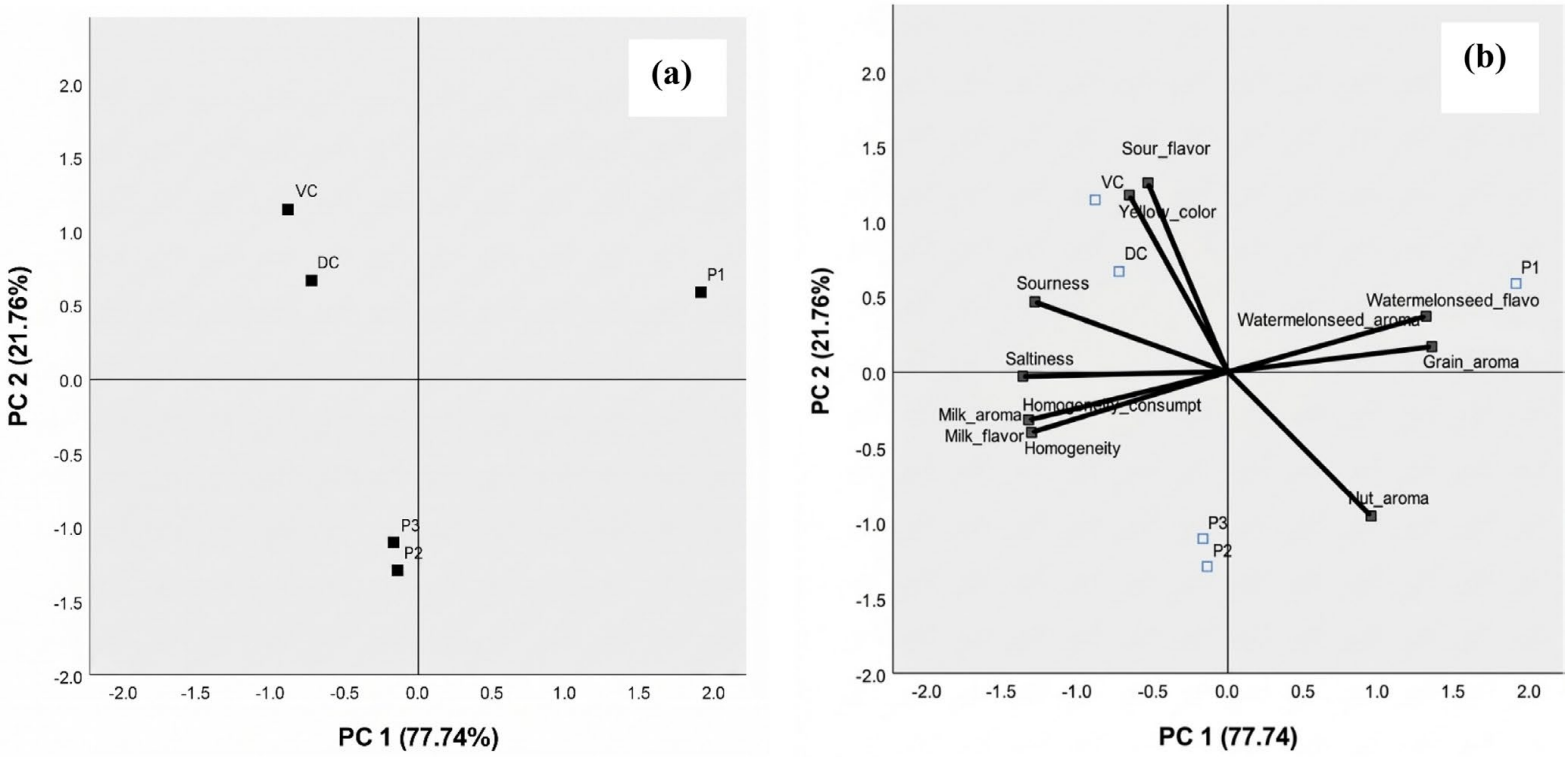
The Principle component analysis (PCA) of variables principle (a) and biplot of sensory attribute evaluated by descriptive analysis (b): DC, dairy cream cheese; P1, prototype; P2, plant‐based dairy (PBD); P3, optimized watermelon seed cream cheese; VC, non‐dairy cream cheese.

A hybrid descriptive analysis method was employed to evaluate the samples. This hybrid approach was chosen to ensure high sensitivity in detecting attribute differences while maintaining consistent intensity scoring against reference standards. The resulting sensory data were further visualized through principal component analysis (PCA), as shown in Figure 4a,b. A total of 79% of the variation was explained by PC1, while 21% was captured by PC2, indicating that most sensory differences among the samples were represented by the first two principal components.

In the PCA biplot, a close association was observed between sample P1 and attributes such as watermelon seed aroma, nut aroma, and grain aroma. These characteristics suggested a strong influence of the raw seed components in the early formulation. This association was found to be consistent with reports by Plamada et al. (2023), which stated that seed‐based dairy analogues often retain pronounced nutty and grain‐like notes before optimization.

As development progressed, a shift toward lower intensities of nut and grain‐related attributes was shown by sample P2. This shift suggested that changes in ingredient ratios were effective in reducing seed‐based notes. These observations were in agreement with studies by Trindler et al. (2022), which showed that raw‐seed aroma expression can be reduced by emulsifier adjustments and heat treatment. Furthermore, a further reduction in watermelon seed, nut, and grain aromas was displayed by the final optimized formulation, P3. In the PCA biplot, P3 was positioned closer to milk aroma and milk flavor, indicating that the sensory profile had moved toward characteristics typically associated with commercial cream cheese. The increased association of P3 with milk‐related attributes showed that dairy‐like notes are often improved in optimized plant‐based formulations after adjustments in fat–water ratios and stabilizer systems.

Both DC and VC were positioned in the same region of the PCA biplot, where they were associated with milk aroma, milk flavor, yellow color, and sour flavor. These attributes are commonly found in cream cheese products and are known to result from lactic acid fermentation and the presence of milk fat (Brighenti et al. 2008). The yellow color observed in both samples was attributed to fat‐soluble pigments derived from dairy fat or plant‐based fat sources (Cheng et al. 2021). The sour flavor was linked to the low pH produced during the acidification process used in cream cheese manufacture. Although DC and VC exhibited distinct differences in taste profile as shown in Figure 2, they shared a similar overall sensory distribution pattern in the PCA model, suggesting that the non‐dairy formulation was produced to closely resemble the sensory properties of conventional cream cheese. DC and VC were therefore used as reference samples in this study. The shift of P3 toward the DC and VC cluster in the PCA biplot indicated that the optimized watermelon seed cream cheese formulation was closer in sensory profile compared to the commercial products compared to the earlier prototypes.

## 4 Conclusion

This study compared the physicochemical properties, volatile compounds, taste profiles, amino acid composition, and sensory characteristics of watermelon seed cream cheese (WCC) with commercial dairy cream cheese (DC) and non‐dairy cream cheese (VC). Differences were found among all samples in all analyses. SEM analysis showed that the microstructure of WCC changed progressively from P1 to P3, where a more compact and porous network was formed with the addition of hydrocolloids, salt, and maltodextrin. The microstructure of P3 was more similar to DC and VC than those of P1 and P2. GC‐E‐Nose analysis identified 30 volatile compounds across seven chemical groups. DC contained high levels of fermentation‐related compounds such as butanoic acid, acetoin, and 2,3‐butanediol, which are linked to cheesy and buttery notes. VC had the lowest volatile levels among all samples. In WCC samples, ethanol and hexanal were highest in P1 and were reduced in P2 and P3 after acidification and matrix stabilization. The volatile profile of P3 was between those of VC and DC. E‐tongue analysis showed that DC had high sweetness and bitterness with low saltiness, while VC had high sourness and umami. P3 had the highest saltiness and moderate sourness, with a taste profile closer to VC than P1 and P2. Amino acid analysis showed that DC had the highest total amino acid content, with glutamic acid and tyrosine as the main components. VC had lower amino acid levels, with phenylalanine and arginine as the main components. WCC samples had arginine‐rich profiles with lower essential amino acid levels than DC. Descriptive sensory analysis showed that P1 was associated with watermelon seed, nut, and grain aromas. These attributes were reduced in P2 and P3, while milk aroma and milk flavor increased. P3 was positioned closer to DC and VC in the PCA biplot, where milk aroma, milk flavor, yellow color, and sour flavor were associated. These results showed that formulation optimization shifted the sensory and physicochemical profile of WCC closer to commercial cream cheese, supporting its potential as a plant‐based alternative. For future research, sensory evaluation should be conducted using a larger and more gender‐balanced panel to improve the representativeness and generalizability of the findings.

## Author Contributions

**Natthanicha Banpacha:** formal analysis. **Gerry Renaldi:** writing – review and editing. **Hiran Janpeng:** conceptualization, data curation, formal analysis, investigation, methodology, resources, software, visualization, writing – original draft. **Rajnibhas Sukeaw Samakradhamrongthai:** project administration, supervision, validation, writing – review and editing.

## Funding

This research project was supported by Fundamental Fund 2025 (207635), Chiang Mai University and also Thailand Science Research and Innovation (TSRI) (FRB680102/0162).

## Ethics Statement

This study was conducted following the guidelines presented in the Declaration of Helsinki. The Office of Human Research Ethics Committee, Chiang Mai University (Approval No. CMUREC 67/072).

## Conflicts of Interest

The authors declare no conflicts of interest.

## Supporting information


**Figure S1:** Appearance of watermelon seed cream cheese analogue: a = prototype WCC, b = PBD WCC, c = optimized WCC.
**Figure S2:** Chromatogram of amino acid standard solution.
**Figure S3:** Chromatogram of dairy cream cheese.
**Figure S4:** Chromatogram of non‐dairy cream cheese.
**Figure S5:** Chromatogram of prototype WCC.
**Figure S6:** Chromatogram of PBD WCC.
**Figure S7:** Chromatogram of optimized WCC.
**Table S1:** The experimental design of watermelon seed milk, salt, and maltodextrin using a mixture design (D‐optimal) with 3 center points for the WCC.

## Data Availability

The data that support the findings of this study are available from the corresponding author upon reasonable request.
