# Supplementary material for: Comparative Study on Morphology, Flavor, and Taste Profile, and Descriptive Sensory Analysis of Conventional Cream Cheese and Non‐Dairy Cream Cheese
Source: Food Sci Nutr. 2026 Sep 2;14(9):e72301. doi: 10.1002/fsn3.72301 (PMC13536484; doi:10.1002/fsn3.72301)
Supplement: Supplementary file 1 [file FSN3-14-e72301-s001.docx]

(c)

(b)

(a)

**Fig. S1.** Appearance of watermelon seed cream cheese analogue: a = prototype WCC, b = PBD WCC, c = optimized WCC

**Fig. S2.** Chromatogram of amino acid standard solution

**Fig. S3.** Chromatogram of dairy cream cheese

**Fig. S4.** Chromatogram of non-dairy cream cheese

**Fig. S5.** Chromatogram of prototype WCC

**Fig. S6.** Chromatogram of PBD WCC

**Fig. S7.** Chromatogram of optimized WCC

**LIST OF ABBREVIATIONS FOR AMINO ACID**

| **Asp** | Aspartic acid |
| --- | --- |
| **Thr** | Threonine |
| **Ser** | Serine |
| **Glu** | Glutamic acid |
| **Pro** | Proline |
| **Gly** | Glycine |
| **Ala + Cys** | Alanine + Cystine |
| **Val** | Valine |
| **Met** | Methionine |
| **Ile** | Isoleucine |
| **Leu** | Leucine |
| **Tyr** | Tyrosine |
| **Phe** | Phenylalanine |
| **His** | Histidine |
| **Lys** | Lysine |
| **Arg** | Arginine |

**Table S1.** The experimental design of watermelon seed milk, salt, and maltodextrin using a mixture design (D-optimal) with 3 center points for the WCC

| **Formulation** | **Watermelon seed milk** | **Salt** | **Maltodextrin** |
| --- | --- | --- | --- |
| **1** | 80.00 | 1.00 | 5.00 |
| **2** | 77.50 | 1.00 | 7.50 |
| **3** | 71.00 | 5.00 | 10.00 |
| **4** | 76.00 | 5.00 | 5.00 |
| **5** | 75.00 | 1.00 | 10.00 |
| **6** | 75.50 | 3.00 | 7.50 |
| **7** | 78.00 | 3.00 | 5.00 |
| **8** | 75.50 | 3.00 | 7.50 |
| **9** | 75.50 | 3.00 | 7.50 |
| **10** | 73.15 | 2.85 | 10.00 |
| **11** | 73.62 | 5.00 | 7.38 |
